# Intranasal delivery of a chimpanzee adenovirus vector expressing a pre-fusion spike (BV-AdCoV-1) protects golden Syrian hamsters against SARS-CoV-2 infection

**DOI:** 10.3389/fcimb.2022.979641

**Published:** 2022-11-03

**Authors:** Shen Wang, Long Xu, Ting Mu, Mian Qin, Ping Zhao, Liang Xie, Linsen Du, Yue Wu, Nicolas Legrand, Karine Mouchain, Guillaume Fichet, Yi Liu, Wenhao Yin, Jin Zhao, Min Ji, Bo Gong, Michel Klein, Ke Wu

**Affiliations:** ^1^ Regularoty and Medical Affairs Department, Wuhan BravoVax Co., Ltd., Wuhan, China; ^2^ Project Management Department, Wuhan BravoVax Co., Ltd., Wuhan, China; ^3^ Innovative Discovery Department, Wuhan BravoVax Co., Ltd., Wuhan, China; ^4^ Test Development Department, Wuhan BravoVax Co., Ltd., Wuhan, China; ^5^ China Office, Voisin Consulting Life Sciences, Shanghai, China; ^6^ In Vivo Sciences Department, Oncodesign, Centre François Hyafil, Villebon-sur-Yvette, France; ^7^ DMPK & Bioanalytical Sciences Department, Oncodesign, Centre François Hyafil, Villebon-sur-Yvette, France; ^8^ In Vitro Sciences Department, Oncodesign, Centre François Hyafil, Villebon-sur-Yvette, France; ^9^ State Key Laboratory of Biocatalysts and Enzyme Engineering, School of Life Sciences, Hubei University, Wuhan, China; ^10^ Executive Office, Wuhan BravoVax Co., Ltd., Wuhan, China; ^11^ Executive Office, Shanghai BravoBio Co., Ltd., Shanghai, China

**Keywords:** Chimpanzee Adenovirus Serotype 68, intranasal, COVID-19 vaccine, challenge study, golden Syrian hamsters

## Abstract

We evaluated the immunogenicity and protective ability of a chimpanzee replication-deficient adenovirus vectored COVID-19 vaccine (BV-AdCoV-1) expressing a stabilized pre-fusion SARS-CoV-2 spike glycoprotein in golden Syrian hamsters. Intranasal administration of BV-AdCoV-1 elicited strong humoral and cellular immunity in the animals. Furthermore, vaccination prevented weight loss, reduced SARS-CoV-2 infectious virus titers in the lungs as well as lung pathology and provided protection against SARS-CoV-2 live challenge. In addition, there was no vaccine-induced enhanced disease nor immunopathological exacerbation in BV-AdCoV-1-vaccinated animals. Furthermore, the vaccine induced cross-neutralizing antibody responses against the ancestral strain and the B.1.617.2, Omicron(BA.1), Omicron(BA.2.75) and Omicron(BA.4/5) variants of concern. These results demonstrate that BV-AdCoV-1 is potentially a promising candidate vaccine to prevent SARS-CoV-2 infection, and to curtail pandemic spread in humans.

## Introduction

Since the end of 2019, more than 600 million people have been infected with SARS-CoV-2 (resource: www.who.int, accessed September 4, 2022), and this number is still increasing as the coronavirus disease 2019 (COVID-19) outbreak and the virus spread globally (Velavan and Meyer, 2020). The approval and emergency usage authorization of COVID-19 vaccines worldwide have been critical in the fight against SARS-CoV-2. So far, the availability of a few licensed COVID-19 vaccines (Huang et al., 2021) has effectively mitigated the pandemic in most countries (Gee et al., 2021; Dagan et al., 2021). In this context, their protective efficacy against COVID-19 has considerably stimulated the interest of the scientific community and the general public.

Among novel approaches to prevent viral infections, the rapidly evolving adenovirus vector-based gene delivery platform has been widely used to engineer vaccines for a wide range of infectious diseases and successfully applied to the production of SARS-CoV-2 vaccines (Koirala et al., 2020). A few recombinant adenovirus-based vaccines have been efficiently administered worldwide and continue to play an important role in controlling and preventing the spread of the pandemic. At present, these vaccines include Convidecia (CanSinoBIO, China), Sputnik V (Gamaleya Institute, Russia), Vaxzevria (Oxford/AstraZeneca,UK), Covishield (India), and AD26.COV2.S (Johnson & Johnson, USA) (Sadoff et al., 2021; Wu et al., 2021; Zare et al., 2021). All adenovirus-based vaccines currently on the market are based on the full-length spike protein (S), are administered intramuscularly and do not induce the mucosal immunity necessary to fully protect against infection and prevent virus shedding and transmission. The SARS-CoV-2 spike which mediates receptor binding and membrane fusion is the primary target for virus neutralization and thus, the immunogen of choice in the design of new-generation COVID-19 vaccines. Viral glycoproteins stabilized in an optimal trimeric prefusion conformation are superior immunogens to their wild-type counterparts (Pallesen et al., 2017; Hsieh et al., 2020). Several vaccines based on SARS-CoV-2 pre-fusion spike have excellent neutralizing activities, including pre-S adjuvanted in ASO3 or alum/CpG (Richmond et al., 2021), delivered with the Ad26 adenovirus vector (Mercado et al., 2020), exposed on nanoparticles (Keech et al., 2020) or encoded by mRNAs (Corbett et al., 2020; Jackson et al., 2020).

Ideally, new-generation vaccines should induce balanced, durable humoral and Th1 T-cell responses as well as mucosal immunity. Mucosal immunization elicits both systemic immune responses and long-lasting protective immunity in the upper and lower respiratory tracts. In contrast to intramuscular vaccination, mucosal immunization is a non-invasive procedure and is the only approach to induce potent and long-lasting secretory IgA responses (sIgA). Robust local immunity at the ports of virus entry should be more efficient at controlling virus infection, replication, shedding and transmission than systemic immunization. Several intranasal and oral vaccines are currently being evaluated in pre-clinical and clinical studies (Kar et al., 2022). They should allow for large-scale vaccination to achieve herd immunity.

The golden Syrian hamster model has been extensively used to study human infectious diseases. *In silico* studies revealed that the angiotensin-converting enzyme 2 (ACE2) receptor of the Syrian hamster is highly homologous to its human counterpart and that it could thus efficiently interact with the SARS-CoV-2 receptor-binding domain (RBD) (Chan et al., 2020; Rosenke et al., 2020) and render animals susceptible to SARS-CoV-2 infection. Indeed, animals challenged intranasally with SARS-CoV-2 isolates consistently showed progressive weight loss, exhibited labored breathing, developed manifestations of disease similar to those of COVID-19 pneumonia observed in humans, as well as other morbidity signs including lethargy, ruffled fur, and hunch posture. Animals may also develop more acute and severe diseases, mimicking severe clinical cases of COVID-19 (O’Donnell et al., 2021; Bednash et al., 2022). Recent results have shown that SARS-CoV can replicate in high titers in both the upper and lower respiratory tracts of infected hamsters that develop pulmonary pathology (Roberts et al., 2005). Thus, golden Syrian hamsters are a valuable model to study the pathogenesis of SARS-CoV-2, evaluate the protective efficacy of COVID-19 vaccines and assess the risk of potential disease enhancement in immune animals challenged with live virus.

We are here reporting that intranasal (IN) delivery of a novel chimpanzee adenovirus vector (Chimpanzee Adenovirus Serotype 68, AdC68) vaccine (BV-AdCoV-1) expressing a stabilized SARS-CoV-2 prefusion spike trimer confers excellent protection in vaccinated golden Syrian hamsters challenged with the ancestral SARS-CoV-2 strain. It also induces cross-neutralizing immunity against the B.1.617.2 (Delta), Omicron (BA.1), Omicron (BA.2.75) and Omicron (BA.4/5) variants of concern (VOCs), indicating that BV-AdCoV-1 could be an efficacious next-generation mucosal COVID-19 vaccine for primary and heterologous booster immunization strategies.

## Materials and methods

### Vector design, construction and production

**Figure 1:**
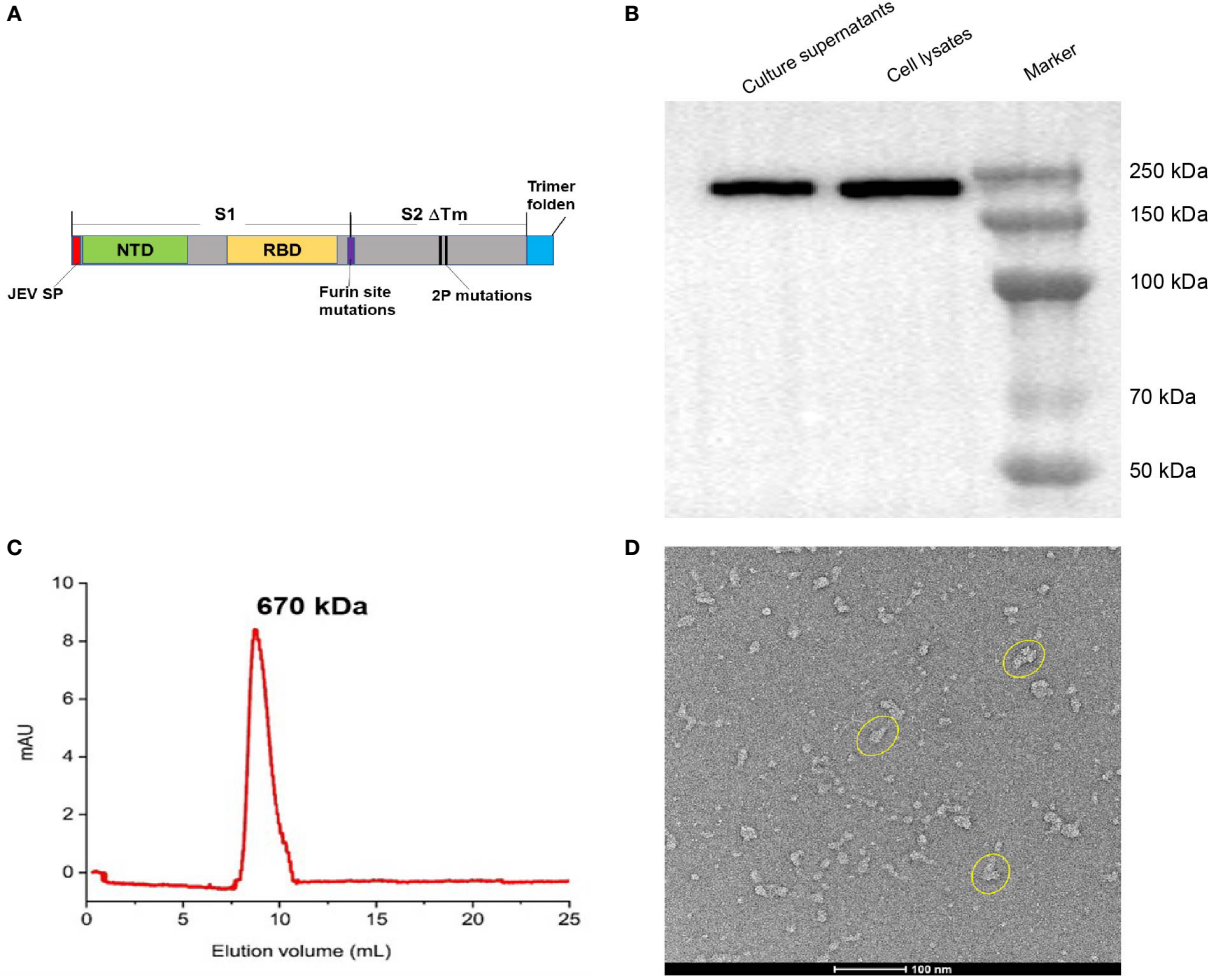
Construction and characterization of BV-AdCoV-1. **(A)** Schematic diagram of the DNA sequence coding for the trimeric prefusion spike (Pre-S). The extracellular domain of SARS-COV-2 spike protein (1-1201 aa) was fused to the T4 fibritin trimerization motif, and the original spike’s signal peptide was replaced by the Japanese encephalitis virus E protein signal peptide (JEV SP). Mutations in the furin cleavage site and proline substitutions (K986P, V987P) were introduced. **(B)** Western blot analysis of HEK 293 cell lysate and supernatant. **(C)** Pre-S protein elution profile on a calibrated Superdex 200 increase 10/300 GL. **(D)** Cryo-EM picture of purified pre-S protein.

The replication-deficient AdC68 derived from the E1/E3 gene-deleted chimpanzee adenovirus type 68 (Suzhou Xiangyi Biotechnology Co., Ltd., China) was engineered to encode a trimeric pre-fusion SARS-CoV-2 spike glycoprotein (pre-S). The spike gene was derived from the SARS-CoV-2 Wuhan-Hu-1 strain. To stabilize the pre-fusion spike conformation (Hsieh et al., 2020), two amino acid residues at positions 986/987 were mutated to prolines (pre-S-2P) and the furin protease cleavage site (residues 682-685) was replaced with GSAS in the wild-type spike glycoprotein (GeneBank ID: MN908947) (**Figure 1A**). The transmembrane and intracellular domains (1202-1273) were replaced by the foldon domain of the Phage T4 fibritin protein and the JEV signal peptide fused to the N-terminal was substituted to the original signal peptide. The codon-optimized DNA fragment encoding the modified spike protein was synthesized by Sangon Biotech Co., Ltd. (China), and cloned into the plasmid pAdC68 backbone. The recombinant plasmid containing the expression cassette pAdC68-PreS harboring the pre-fusion spike gene was linearized and transfected into HEK293A cells (Invitrogen, R70507) to rescue the recombinant adenovirus vector (rAdC68-PreS). HEK293A cells were cultured, grown and maintained in DMEM (Gibco, 12100-061) supplemented with 10% FBS in an incubator at 37 °C in 5% CO_2_. After plaque purification, the recombinant adenovirus was propagated in HEK293A suspension cell cultures and purified by ion-exchange and size-exclusion chromatography (SEC) to produce BV-AdCoV-1. The number of virus particle (VP) was measured by ultraviolet spectrophotometry, and infectious titers were assessed by immunocytochemistry (ICC).

### Expression of trimeric pre-S-2P

To confirm pre-S expression, cell lysates and culture supernatants were mixed with reducing sample buffer [0.25 M Tris HCl (pH 6.8), 40% glycerol, 8% SDS, 5% 2-mercaptoethanol and 0.04% bromophenol blue] and boiled for 10 minutes at 65°C. Proteins resolved by SDS-polyacrylamide gel electrophoresis were transferred to polyvinylidene fluoride membranes, blocked with 5% non-fat powdered milk in PBST (0.5% Tween-20) and probed with rabbit antibodies against SARS-CoV-2 spike RBD (1:2000, Sino Biological, China) at 4°C overnight. Goat Horseradish Peroxidase (HRP)-conjugated secondary antibody (1:2000, BBI, China) was then added for 1 hour at 37°C. Protein bands were detected using Tanon 5200 Chemiluminescent Imaging System (Tanon, China). Quantity One software was used to calculate molecular weights.

Culture supernatants were further concentrated and purified by anion-exchange chromatography (HiTrap Capto Q 5 × 5mL, Cytiva) followed by size-exclusion chromatography (Superdex 200 increase 10/300 GL, Cytiva) using 0.01 M phosphate buffer, 0.14 M NaCl, pH 7.4 as elution buffer, Purified pre-S was submitted to cryo-electron microscopy (Cryo-EM), and SEC-HPLC analysis. The molecular weight of trimeric pre-S was calculated according to the retention time in the column using Origin software. The purified pre-S protein was negatively stained, deposited onto a copper grid and imaged by 120 kV Cryo-EM (Talos L120C G2).

### Immunization and live virus challenge of golden Syrian hamsters

The challenge study in golden Syrian hamsters was performed in a Biosafety Level 3 (BSL-3) laboratory at Oncodesign Biotechnology (France), the animal facility (CFH: Agreement N° B91962106) and BSL-3 facility (Agreement N° D92-032-02). Animal housing and experimental procedures were conducted according to the French and European Regulations and the National Research Council Guide for the Care and Use of Laboratory Animals. All animal procedures (including surgery, anesthesia and euthanasia as applicable) used in the current study were submitted to the Institutional Animal Care and Use Committee of Oncodesign (CNREEA Agreement N° 91) and the CEA (Commissariat à l’Energie Atomique et aux Energies Alternatives Paris-Saclay; Agreement No. CETEA DSV n° 44) approved by French authorities.

**Figure 2:**
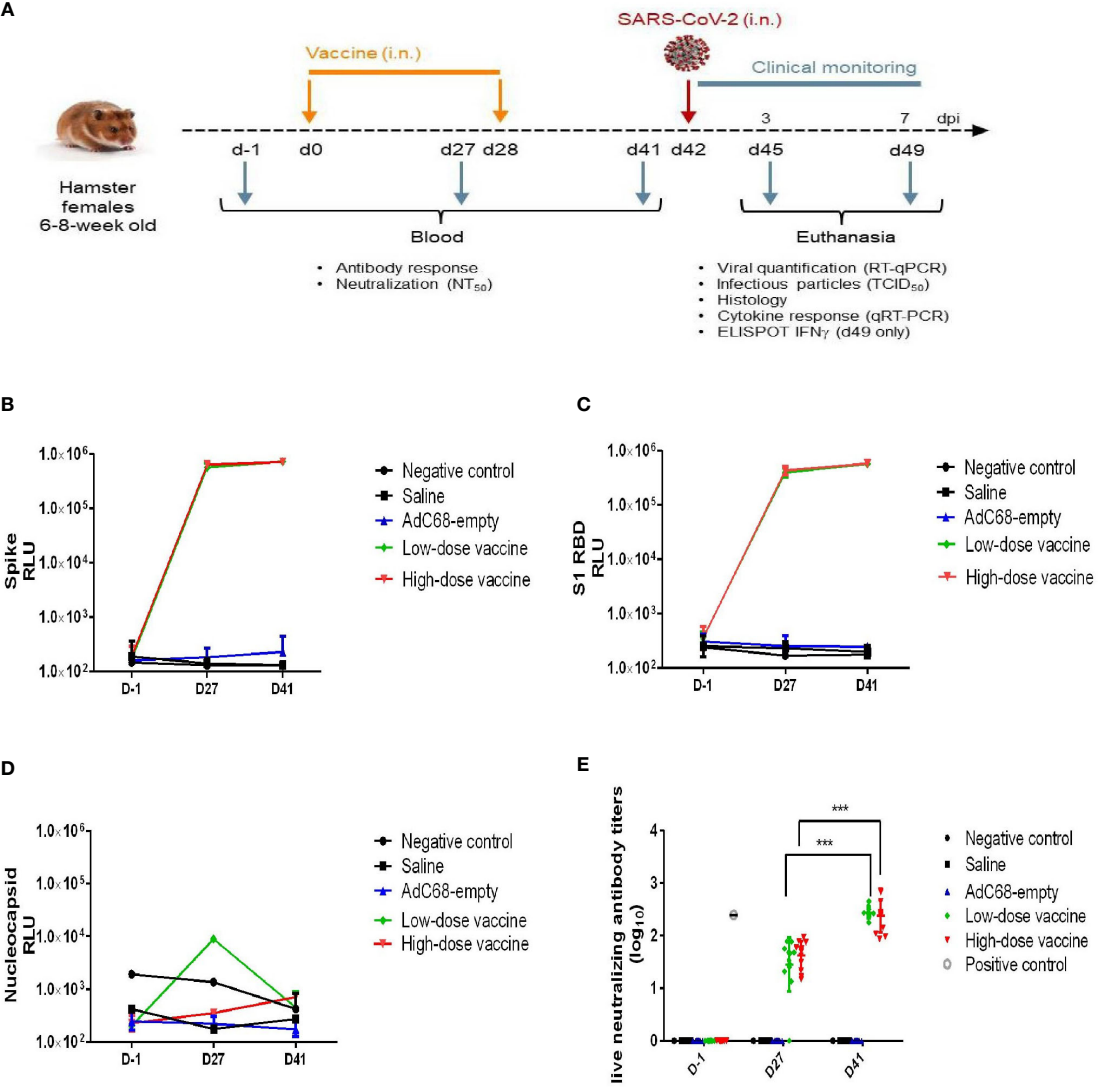
Experimental study scheme and anti-SARS-CoV-2 antibody detection. **(A)** Schedule of vaccine administration and virus challenge. dpi (days post-infection). **(B)** Binding anti-spike IgG antibody in golden Syrian hamsters’ sera. The antibody level was determined by Multiplex ELISA. **(C)** Binding anti-S1 RBD IgG antibody in golden Syrian hamsters’ sera. The antibody levels were determined by Multiplex ELISA. **(D)** Anti-nucleocapsid IgG antibody in golden Syrian hamsters’ sera. The antibody level was determined by Multiplex ELISA. **(E)** Live neutralizing antibody titers in golden Syrian hamsters’ sera. The neutralizing antibody level was determined by live SARS-CoV-2 cytopathogenicity-based assay. ***p < 0.001.

Female golden Syrian hamsters at 6-8 weeks of age were purchased from Janvier Labs (France). Animals were randomized into 4 homogeneous groups (n=12) and one negative control group (3 hamsters, non-treated, not infected) according to weight. The 4 homogeneous groups included a Saline intranasal administration group (12 hamsters, Saline), an AdC68-empty vector intranasal administration group (3.4×10^10^ VP/dose, 12 hamsters, AdC68-empty, vector control), a low-dose BV-AdCoV-1 intranasal administration group (3.4×10^9^ VP/dose, 12 hamsters, low-dose vaccine), and a high-dose BV-AdCoV-1 intranasal administration group (3.4×10^10^ VP/dose, 12 hamsters, high-dose vaccine). The details about grouping and vaccination schedule of golden Syrian hamsters are shown in **Table 1**. The challenge dose for each animal was 10^5^ plaque-forming units (PFU) of SARS-CoV-2 (Slovakia/SK-BMC5/2020 isolate which contained the D614G mutation was provided by the European Virus Archive global [EVAg]–GISAIDEPI_ISL_417879, https://www.european-virus-archive.com/virus/sars-cov-2-strain-slovakiask-bmc52020), in a volume of 70 µL (35 µL per nostril). Among the 12 animals in the Saline, AdC68-empty, low-dose vaccine and high-dose vaccine groups, 6 animals were sacrificed 3 days post-challenge, while the remaining 6 animals were sacrificed 7 days post-challenge. The schedule of vaccine administration and virus challenge is shown in **Figure 2A**.

**Table 1:** Grouping of golden Syrian hamsters and vaccine dose.

Group	No. animals	Test	Injections (intranasal)	Dosage/injection	End point
Negative control	3	–	NA	NA	D49
Saline	6	Saline	NA	NA/70 µL	D45
	6				D49
AdC68-empty	6	AdC68-empty	D0, D28	3.36×10^10^VP/70 µL	D45
	6				D49
Low-dose vaccine	6	BV-AdCoV-1	D0, D28	3.40×10^9^ VP/70 µL	D45
	6				D49
High-dose vaccine	6	BV-AdCoV-1	D0, D28	3.40×10^10^VP/70 µL	D45
	6				D49

To explore the cross-neutralizing activity of BV-AdCoV-1 immune sera against VOCs, another immunogenicity study was conducted in China.

Female golden Syrian hamsters at 6-8 weeks of age were purchased from Beijing Vital River Laboratory Animal Technology Co., Ltd. (China). The immunogenicity study was conducted in the Wuhan Myhalic Biotechnological Co., Ltd. (China). The vaccination protocol was approved by the Animal Ethics Committee (No. HLK-20220630-001 for hamsters) of Wuhan Myhalic Biotechnological Co., Ltd.

**Figure 3:**
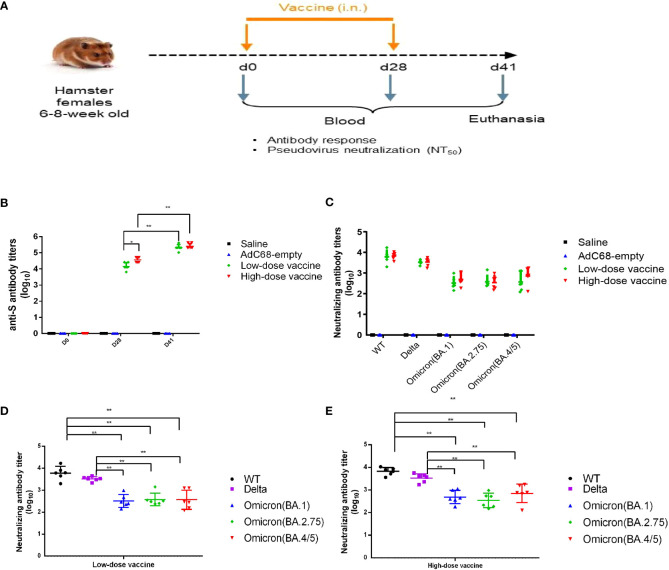
Experimental study scheme and cross-neutralizing antibody titers. **(A)** Schedule of vaccine administration. **(B)** Binding anti-spike IgG antibody titers in golden Syrian hamsters’ sera. **(C–E)** Cross-neutralizing antibody titers in golden Syrian hamsters’ sera. *p < 0.05, **p < 0.01.

Animals were randomized into 4 homogeneous groups (n=6). The 4 homogeneous groups included a Saline intranasal administration group (6 hamsters, Saline), an AdC68-empty vector intranasal administration group (3.4×10^10^ VP/dose, 6 hamsters, AdC68-empty, vector control), a low-dose BV-AdCoV-1 intranasal administration group (3.4×10^9^ VP/dose, 6 hamsters, low-dose vaccine), and a high-dose BV-AdCoV-1 intranasal administration group (3.4×10^10^ VP/dose, 6 hamsters, high-dose vaccine). The details about grouping and vaccination schedule of golden Syrian hamsters are shown in **Table 2** and **Figure 3A**.

**Table 2:** Grouping of golden Syrian hamsters and vaccine dose.

Group	No. animals	Test	Injections (intranasal)	Dosage/injection
Saline	6	Saline	NA	NA/70 µL
AdC68-empty	6	AdC68-empty	D0, D28	3.40×10^10^VP/70 µL
Low-dose vaccine	6	BV-AdCoV-1	D0, D28	3.40×10^9^ VP/70 µL
High-dose vaccine	6	BV-AdCoV-1	D0, D28	3.40×10^10^VP/70 µL

### Binding antibody titers determination by Multiplex ELISA in the challenge study

Binding IgG responses against SARS-CoV-2 in hamster sera were analyzed using V-PLEX SARS-CoV-2 Panel 2 plates from Meso Scale Discovery (kit K15383U, USA). Responses to the S, S1 RBD and N antigens were quantified using a multiplex approach. MULTI-SPOT plates were provided with antigens coated on spots in the wells of a 96-well plate. Antibodies bound to the spots were detected using goat anti-Syrian hamster IgG (H and L chains) antibodies (Abcam, reference ab102314) conjugated with MSD SULFO-TAG using the MSD GOLD™ SULFO-TAG TM NHS-Ester kit (MSD, reference R91AO-1). The plates were read on a MESO Quickplex SQ120 imager that measured the light emitted from the MSD SULFO-TAG, and the results were analyzed using the associated software (Discovery Workbench).

### Live virus neutralization assay in the challenge study

Sera were inactivated at 56°C for 30 minutes before the neutralization assay. They were diluted with DMEM medium containing 2% FBS at an initial dilution of 1:5 (v:v), then three-fold diluted to perform the assay. Samples were then mixed (1:1, v:v) with 0.01 MOI per well of SARS-CoV-2 virus (Slovakia/SK-BMC5/2020 isolate) for 30 minutes at room temperature to allow antibody binding to the virus. The virus-antibody mixture (50 µL) was added to Vero E6-TMPRSS2 cells (2×10^4^ cells/well) in 200 µL complete growth medium, and further incubated for 2 hours at 37°C, 5% CO_2_ to allow viruses to infect target cells. Subsequently, 150 µL of complete cell growth medium (containing 2% FBS) were added to each well. The plates were further incubated for 48 hours at 37°C, in 5% CO_2_. After removing 100 µL of supernatant from each well, 100 µL of fresh medium and 20 µL of MTS-PMS reagent (Cell Titer 96^®^AQueous Non-Radioactive Cell Proliferation Assay, Promega reference G5421) were added for colorimetric determination of the viable cell number. Plates were read using an ELISA plate reader, and data were recorded (acceptance criteria when OD_450nm_ of control cells >1.5). The neutralizing antibody titer (NT_50_) was defined as the reciprocal of the highest serum dilution that provided ≥50% inhibition of virus infectivity. The NIBSC pooled convalescent serum reference (WHO international standard for anti-SARS-CoV-2 human immunoglobulin, NIBSC reference 20/136) was used as positive control.

### Binding antibody titers determination by ELISA in the immunogenicity study

Binding IgG titers in immune sera were measured by ELISA. 96-well plates were coated with 0.05 µg of full-length S protein (Genscript Inc., China) overnight at 4°C and blocked with phosphate-buffered saline (PBS) supplemented with 0.05% Tween-20 (PBST) and 10% NON-Fat Powdered Milk for 2 hours at 37 °C. The microplates were then washed three times with PBST. Sera samples were serially diluted 2-fold and added to the wells. After further incubation for 1 hour at 37°C, plates were washed three times with PBST and 100µL of horseradish peroxidase (HRP)-conjugated goat anti-hamster IgG antibody (1:5000; abcam, ab6892) was added to wells. After incubation for another 1 hour at 37°C, plates were washed three times with PBST and then 100µL of 3,3’,5,5 -tetramethylbenzidine (TMB) substrate was added to wells. Following a 12-15 minutes incubation at room temperature in the dark, reactions were stopped with 100µL of 1M sulfuric acid. The optical density was measured at 450 nm. The cutoff value was calculated as 2.1 times the mean OD_450 nm_ values obtained for samples from non-vaccinated animals. The endpoint titer was calculated as the reciprocal of the highest sample dilution at which the OD_450 nm_ value was equal to or greater than the cutoff value.

### Pseudovirus neutralizing antibody titers determination in the immunogenicity study

Sera were inactivated at 56°C for 30 minutes, 4-fold serially diluted with cell culture medium and added to a 96-well plate at 100 µL/well. Pseudoviruses at 1.3×10^4^ TCID_50_/mL were added (50 µL/well) to the plate. The mixtures were incubated at 37°C, 5% CO_2_ for 1 hour, along with a negative control and a virus control. 100 µL of Vero cells were added to the 96-well plate at 2×10^4^ cells/well. Plates were incubated at 37°C, 5% CO_2_ for 24 hours. Finally, the plates were analyzed according to the luciferase assay kit instructions (PerkinElmer, 6066761). The Reed-Muench method was used to calculate the 50% inhibitory concentration (IC_50_) value.

Pseudoviruses included SARS-CoV-2 ancestral pseudovirus (WT), B.1.617.2 (Delta), Omicron (BA.1), Omicron (BA.2.75) and Omicron (BA.4/5) pseudoviruses. They were purchased from Beijing Yunling Biotechnology Co., Ltd. (China), company related to the China National Institutes for Food and Drug Control.

### Enzyme-linked immunospot assay (ELISpot)

T-cell responses against SARS-CoV-2 antigens were evaluated in a hamster ELISpot IFN-γ assay (Mabtech, 3102-2A). The kit was used according to the manufacturer’s instructions. Briefly, live immune splenocytes were isolated, counted and re-stimulated with specific 15-mer peptide pools with 11 amino-acid overlap (JPT; PepMix SARS-COV2 S-RBD, reference PM-WCPV-S-RBD-2; PepMix SARS-COV-2 Spike, reference PM-WCPV-S-2; PepMix SARS-COV2 NCAP, reference PM-WCPV-NCAP-2). The ELISpot assay was performed in triplicates at two cell densities (200×10^3^ and 60×10^3^ cells per well), comparing different conditions: medium only; PMA (20 ng/mL) and ionomycin (1 µM) mix as positive control; peptide pool (2 µg/mL). A lower cell density (10×10^3^ cells per well) was used for the PMA/ionomycin positive control. Plates were placed in an incubator at 37 °C in 5% CO_2_ for 24 hours after which time cells were removed, and IFN-γ-producing cells were detected as recommended by the manufacturer.

### Virus load determination in lung homogenates by RT-qPCR

The extraction of total RNA and its conversion to cDNA were conducted according to the manufacturer’s instructions (Macherey nagel Nucleo Spin 96 RNA, 96-well kit, Applied Biosystem Kit). The number of relative levels of RNA (target gene cycle threshold [Ct] value) in the lungs was determined by real-time quantitative PCR (RT-qPCR) after reverse transcription. Quantification of viral loads by RT-qPCR was done using the ORF1ab gene. The primer set for ORF1ab was 5’-CCGCAAGGTTCTTCTTCGTAAG-3’, and the probe set for ORF1ab was 5’-TGCTATGTTTAGTGTTCCAGTTTTC-3’. SYBR Green technology was used for PCR product detection and quantification. Results are expressed as a 2^-ΔCt^ value relative to the γ-actin house-keeping gene for normalization between samples.

### Cytokine response profiling by RT-qPCR

Cytokine gene expression in the lungs was determined for 9 target genes: TNF-α, IFN-γ, IL-2, IL-4, IL-5, IL-6, IL-10, IL-12p40, IL-21. The extraction of total RNA and its conversion to cDNA were conducted according to the manufacturer’s instructions (NucleoSpin 96 RNA, 96-well kit, Applied Biosystem Kit). cDNA quantification (target gene Ct value) by real-time quantitative PCR was performed with primers targeting the cytokine genes (**Table 3**). Amplification was performed using a QuantStudio 7 Flex from Applied Biosystem and adjoining software. SYBR Green technology was used for PCR product detection and quantification. Results are expressed as a 2^-ΔCt^ value relative to the γ-actin house-keeping gene for normalization between samples.

**Table 3:** Primer sequences for each target cytokine gene.

Primers and probes	
Name	Sequences (5’-3’)
TNF-α	Fw : TGAGCCATCGTGCCAATG Rv : AGCCCGTCTGCTGGTATCAC
IFN-γ	Fw : TGTTGCTCTGCCTCACTCAGG Rv : AAGACGAGGTCCCCTCCATTC
IL-2	Fw :CCAGTGCCTGGAAGAAGAACTT Rv : CATCTTCCAAGTGAAAGCTTTTGCT
IL-4	Fw :ACAGAAAAAGGGACACCATGCA Rv : GAAGCCCTGCAGATGAGGTCT
IL-5	Fw : GTTCCTGCACATAAAAATCACC Rv : AACTGCTTCACTCTCCGTC
IL-6	Fw : AGACAAAGCCAGAGTCATT Rv : TCGGTATGCTAAGGCACAG
IL-10	Fw : GGTTGCCAAACCTTATCAGAAATG Rv : TTCACCTGTTCCACAGCCTTG
IL-12p40	Fw : AATGCGAGGCAG CAAATTACTC Rv : CTGCTCTTGACGTTGAACTTCAAG
IL-21	Fw : GGACAGTGGCCCATA AAACAAG Rv : TTCAACACTGTCTATAAGATGACGAAGTC

### Virus 50% tissue culture infectious dose (TCID_50_) determination

According to L. J. Reed (Reed and Muench, 1938), Vero E6/TMPRSS2 cells were plated at a density of 2×10^4^ cells per well in 200 µL of complete growth medium. Cells were then infected with serial dilutions of the lung homogenates in duplicate for 1 hour at 37°C and fresh medium was added. Two days after cell infection, an MTS/PMS assay was performed according to the manufacturer’s instructions (Promega, G5430). TCID_50_ was defined as the amount of pathogen that caused the death of 50% of target cells. Infectivity was expressed as TCID_50_/g of lungs (over 48-hour culture) based on the Spearman-Karber formula. The lower limit of detection of the assay is 520 TCID_50_/g of lung tissue.

### Lung histopathology

Left lung lobe specimens were embedded in paraffin. 5 µm-thick sections were cut, mounted on SuperFrost plus glass slides and stained with Hematoxylin-Phloxine to visualize histomorphometric changes. Slides were scanned using the NanoZoomer Digital Pathology System C9600-02.

Histopathology scores were determined for each one of the four sections generated from the collected lobe, as an aggregate of the following parameters: (i) percentage of tissue area showing signs of inflammation (presence of inflammatory leucocyte infiltrates): 0, no pathological change; 1, ≤10% affected area; 2, >10% to <50% affected area; 3, ≥50% affected area; (ii) pulmonary edema (0: absent; 1: present); and (iii) alveolar hemorrhage (0: absent; 1: present) (Imai et al., 2020). The average score for four sections is shown for each individual animal.

### Statistical analysis

The data were processed using the Graphpad software. Results reported in this study are expressed as means ± standard deviation (SD). Differences were identified using ANOVA and considered to be significant when *p <*0.05, 0.01, 0.001 and 0.0001.

## Results

### Generation and characterization of BV-AdCoV-1

We constructed a recombinant AdC68-vectored vaccine (BV-AdCoV-1) to express a trimeric pre-fusion spike in infected cells. The pre-fusion conformation was stabilized by S-2P mutations and the furin cleavage site was mutated. Deletion of the transmembrane and intracellular domains facilitated the secretion of the immunogen. The foldon domain caused the secreted spike protein to form a trimer, in a conformation similar to that of its native state on the surface of the virion. BV-AdCoV-1 was successfully rescued and propagated in HEK293A cell line. Cell lysates and culture supernatants analyzed by SDS PAGE under reducing conditions followed by Western blotting revealed the presence of a 222 kDa monomeric pre-S protein (**Figure 1B** and **Supplementary Material 1**). Pre-S was further purified from the culture supernatants of HEK293A cells and shown to be a homogeneous trimer with a molecular mass of 670 kDa according to the retention time measured by analytical size-exclusion chromatography (**Figure 1C**). The pre-S protein was negatively stained and deposited onto a copper grid and observed by 120 kV Cryo-EM (**Figure 1D**). Cryo-EM analysis also showed that pre-S formed trimers.

### BV-AdCoV-1 elicits robust antibody responses

In the challenge study, BV-AdCoV-1 at both low and high dose elicited very high levels of anti-S, anti-S1 RBD binding IgG antibody responses (**Figures 2B, C**) on 27 days after a single intranasal administration, while background levels of anti-nucleocapsid binding IgG antibody responses were low in all golden Syrian hamsters (**Figure 2D**). Binding antibody titers among the two groups did not differ significantly, which may be related to the high immunogen doses (≥ 3.4×10^9^ VP/dose) used for golden Syrian hamsters. Geometric means titer (GMT) of neutralizing antibody titers for the low- and high-dose vaccine groups were 239 and 276, respectively, 41 days after the first vaccination, while the neutralizing antibody titer of the NIBSC reference was 251 (**Figure 2E**). The neutralizing antibody responses to the low and high vaccine doses were similar and both increased significantly (*p <*0.001) after the booster administration.

In the immunogenicity study, the levels of anti-S binding IgG antibody titers were very high in the low-dose and high-dose vaccine groups and could be further boosted. There was no significant difference (*p >*0.05) between the low- and high-dose vaccine groups after a second administration (**Figure 3B**). Immune sera from the low- and high-dose vaccine groups had high levels of neutralizing antibody titers against pseudoviruses of WT and Delta strains, although neutralizing titers against the Delta strain were slightly lower. GMT of pseudovirus neutralizing antibody titers against WT for the low- and high-dose vaccine groups were 6045 and 6721, respectively, while GMT of neutralizing antibody titers against the Delta pseudovirus were 3289 and 3400, respectively, 41 days after the first vaccination. The neutralizing antibody titers against Omicron (BA.1), Omicron (BA.2.75) and Omicron (BA.4/5) pseudoviruses were 10-20-fold and 4-10-fold reduced (*p <*0.01) but still substantial, compared to those against the WT and Delta strains, respectively (**Figures 3B–E**).

### Cellular immunity induced by BV-AdCoV-1

**Figure 4:**
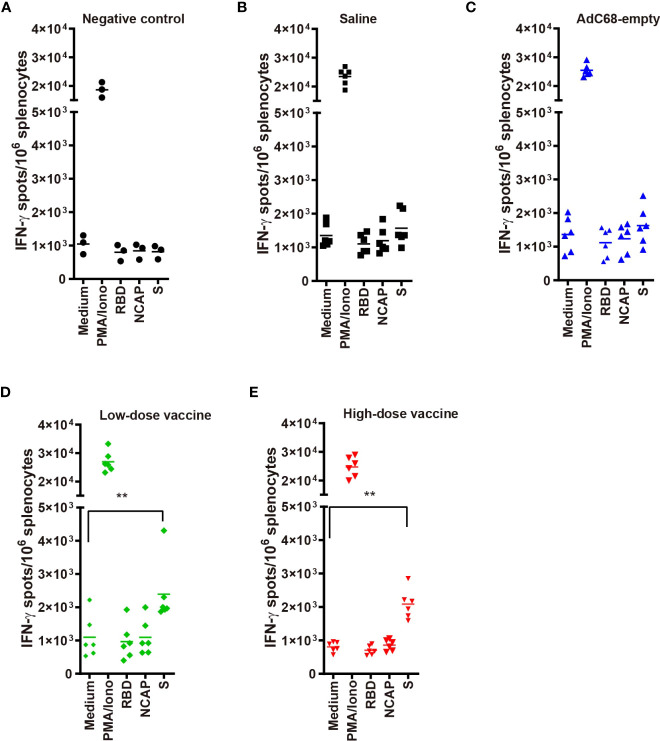
**(A–E)** IFN-γ ELISpots in golden Syrian hamsters’ splenocytes re-stimulated with: medium-only (negative control); PMA and ionomycin (positive control); and peptide pools from RBD, nucleocapsid (N) and spike (S). T-cell responses against SARS-CoV-2 antigens were evaluated using a hamster ELISpot IFN-γ kit at dpi 7. **p<0.01.

In the low- and high-dose vaccine groups (dpi 7, days post-infection 7), IFN-γ signals were significantly higher when immune splenocytes were stimulated with a spike peptide cocktail (*p <*0.01) whereas the IFN-γ signal did not significantly increase when splenocytes were stimulated with either RBD or N peptides compared with medium (**Figure 4**). In contrast, IFN-γ signals after stimulation with a spike peptide cocktail were similar to that of medium in the Negative control, Saline and AdC68-empty groups. The differences observed between responses to spike and RBD peptide pools could be explained by the lack of strong RBD T-cell epitopes recognized by Syrian hamster MHC haplotypes. The N peptide pool served as a negative control.

### Clinical disease in golden Syrian hamsters

**Figure 5:**
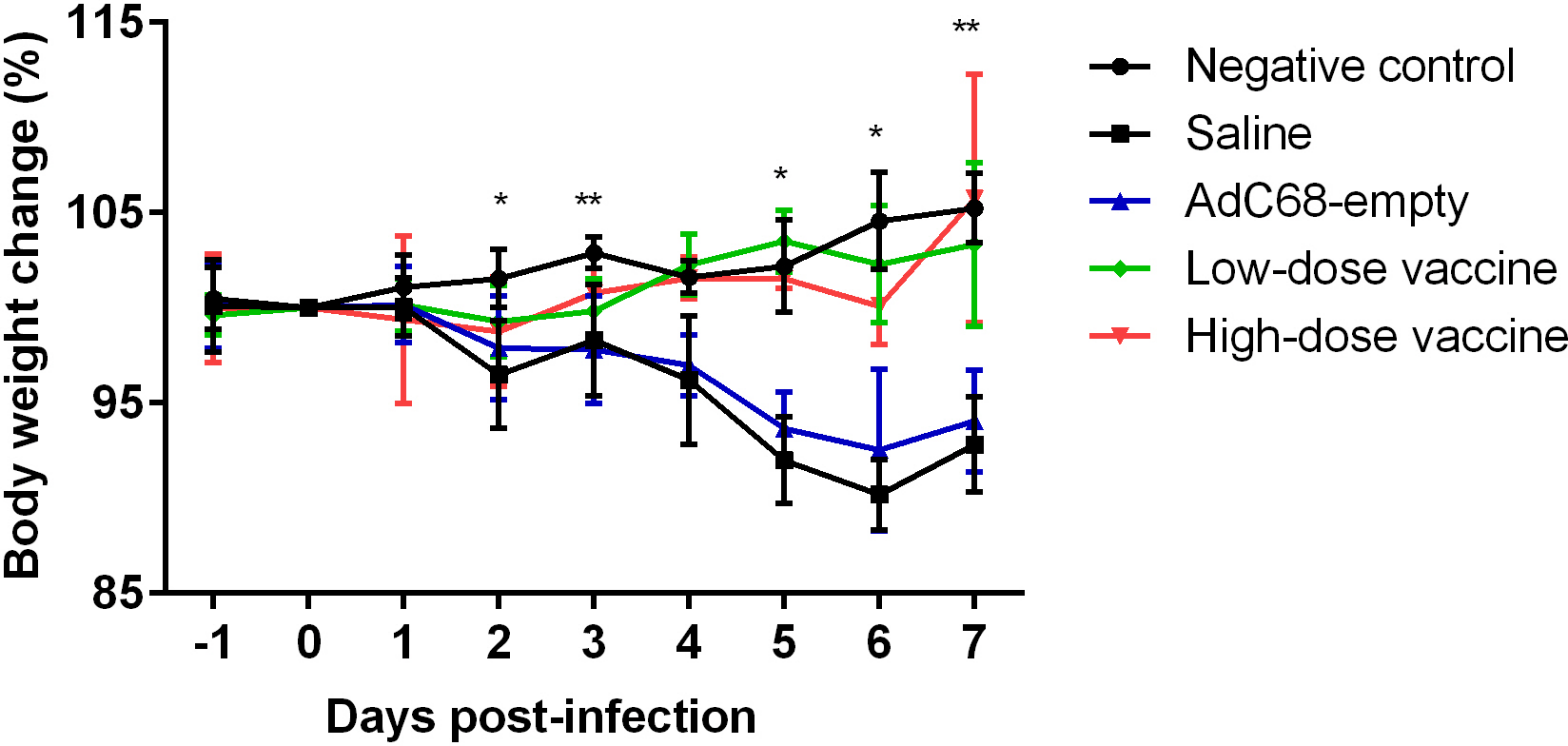
Body weight changes in golden Syrian hamsters after live challenge. Data presented as mean ± SD of values. *P<0.05 or **P<0.01 between Negative control and Saline. Intergroup statistical analysis was performed using a two-way ANOVA test, using the infected, saline-treated group as a reference.

Golden Syrian hamsters were infected with 10^5^ PFU of SARS-CoV-2 and inspected daily for up to 7 days. Changes in body weight (BWC) decreased in the Saline and AdC68-empty groups from 2 days post infection (dpi 2). Comparative analysis of the negative control group and infected Saline-treated animals clearly showed that infection led to significant body weight losses from days 2 to 7 post infection (dpi 2, dpi 3 and dpi 5 to dpi 7) and those hamsters did not even recover their original weight 7 days post infection. In marked contrast, the BWC pattern in animals vaccinated with BV-AdCoV-1 was similar to that of the negative control group, with body weight changes of ≥100% from dpi 3 to dpi 7 (**Figure 5**). These data clearly showed that immunization with BV-AdCoV-1 prevented weight loss due to SARS-CoV-2 infection.

### Protection against SARS-CoV-2 disease

**Figure 6:**
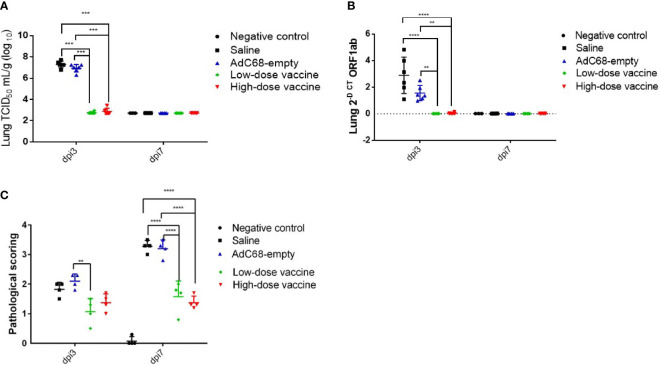
Intranasal BV-AdCoV-1 vaccination protects golden Syrian hamsters from SARS-CoV-2 infection. **(A)** SARS-CoV-2 virus infection titers in the lungs of golden Syrian hamsters were defined as the virus 50% tissue culture infectious dose (TCID_50_). **(B)** SARS-CoV-2 relative levels of RNA in the lungs of golden Syrian hamsters. **(C)** Lung histopathology scores in golden Syrian hamsters. Left lung slides were stained with Hematoxylin-Phloxine to visualize histomorphometric changes. Slides were scanned using the NanoZoomer Digital Pathology System C9600-02. **p < 0.01, ***p < 0.001, ****p < 0.0001.

To assess the protective ability of BV-AdCoV-1 against SARS-CoV-2 disease, relative levels of RNA in the lungs, lung virus infectious doses (TCID_50_) and lung histopathology were examined at dpi 3 and dpi 7. At dpi 3, mean TCID_50_ was similar between the Saline and AdC68-empty groups. In contrast, mean TCID_50_ was dramatically decreased in both the high-dose and low-dose vaccine groups (947 and 583 TCID_50_/g, respectively), representing a 10^5^-10^4^-fold reduction. This result also indicates that the immune response had already plateaued at the lowest vector dose. Mean TCID_50_ reached a baseline level of 520 TCID_50_/g in all groups at dpi 7 (**Figure 6A**). At dpi 3, mean viral levels of ORF1 RNA in the high-dose vaccine group (0.04 2^-Δct^) and low-dose vaccine group (0.01 2^-Δct^) were similar and significantly lower (*p <*0.01) than those in the Saline and AdC68-empty groups. Mean expression reached a baseline level of ≤0.01 2^-Δct^ in all groups at dpi 7 (**Figure 6B**).

**Figure 7:**
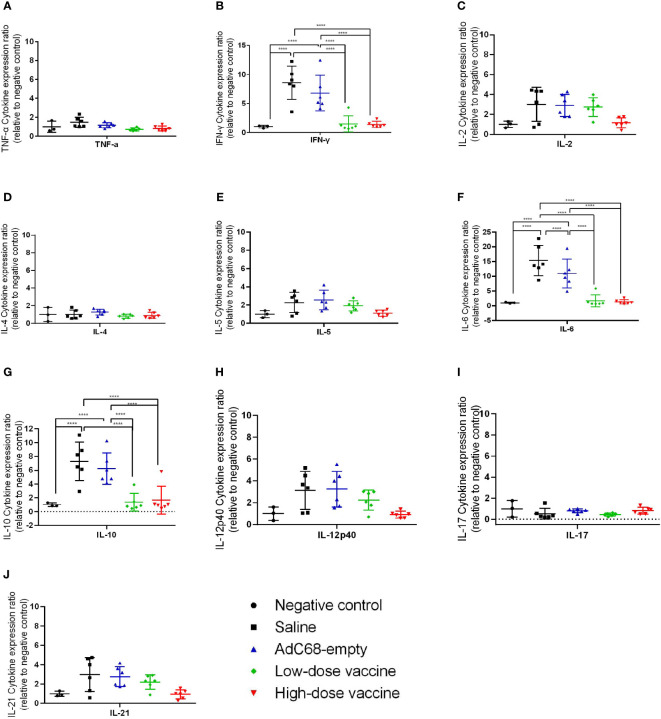
Ratios of cytokine expression levels in the lungs of golden Syrian hamsters at dpi 3. The cytokine expression levels in the lungs of golden Syrian hamsters were determined by RT-qPCR at dpi 3. **(A)** Ratios of TNF-α cytokine expression levels in the lungs of golden Syrian hamsters at dpi 3, **(B)** Ratios of IFN-γ cytokine expression levels in the lungs of golden Syrian hamsters at dpi 3, **(C)** Ratios of IL-2 cytokine expression levels in the lungs of golden Syrian hamsters at dpi 3, **(D)** Ratios of IL-4 cytokine expression levels in the lungs of golden Syrian hamsters at dpi 3, **(E)** Ratios of IL-5 cytokine expression levels in the lungs of golden Syrian hamsters at dpi 3, **(F)** Ratios of IL-6 cytokine expression levels in the lungs of golden Syrian hamsters at dpi 3, **(G)** Ratios of IL-10 cytokine expression levels in the lungs of golden Syrian hamsters at dpi 3, **(H)**Ratios of IL-12p40 cytokine expression levels in the lungs of golden Syrian hamsters at dpi 3, **(I)** Ratios of IL-17 cytokine expression levels in the lungs of golden Syrian hamsters at dpi 3, **(J)** Ratios of IL-21 cytokine expression levels in the lungs of golden Syrian hamsters at dpi 3. ****p < 0.0001.

**Figure 8:**
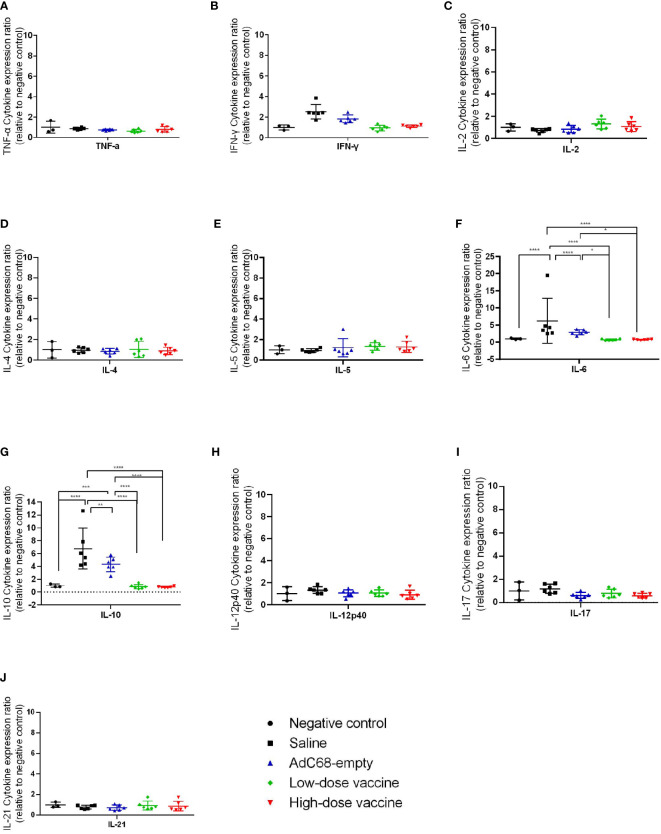
Ratios of cytokine expression levels in the lungs of golden Syrian hamsters at dpi 7. The cytokine expression levels in the lungs of golden Syrian hamsters were determined by RT-qPCR at dpi 7. **(A)** Ratios of TNF-α cytokine expression levels in the lungs of golden Syrian hamsters at dpi 7, **(B)** Ratios of IFN-γ cytokine expression levels in the lungs of golden Syrian hamsters at dpi 7, **(C)** Ratios of IL-2 cytokine expression levels in the lungs of golden Syrian hamsters at dpi 7, **(D)** Ratios of IL-4 cytokine expression levels in the lungs of golden Syrian hamsters at dpi 7, **(E)** Ratios of IL-5 cytokine expression levels in the lungs of golden Syrian hamsters at dpi 7, **(F)** Ratios of IL-6 cytokine expression levels in the lungs of golden Syrian hamsters at dpi 7, **(G)** Ratios of IL-10 cytokine expression levels in the lungs of golden Syrian hamsters at dpi 7, **(H)** Ratios of IL-12p40 cytokine expression levels in the lungs of golden Syrian hamsters at dpi 7, **(I)** Ratios of IL-17 cytokine expression levels in the lungs of golden Syrian hamsters at dpi 7, **(J)** Ratios of IL-21 cytokine expression levels in the lungs of golden Syrian hamsters at dpi 7. *p < 0.05, **p < 0.01, ***p < 0.001, ****p < 0.0001.

We next assessed the effect of BV-AdCoV-1 on lung inflammation and disease. Several proinflammatory cytokines (TNF-α, IFN-γ, IL-2, IL-4, IL-5, IL-6, IL-10, IL-12p40, IL-17, IL-21) RNA levels were measured in lungs at dpi 3 and dpi 7 (**Figures 7**, **8**). The results were expressed as the relative mean cytokine expression level for each group, as compared to the negative control group. Infection with SARS-CoV-2 induced a significant increase in IFN-γ (*p <*0.0001), IL-6 (*p <*0.0001) and IL-10 (*p <*0.0001) expression in the lung tissue of the Saline group and AdC68-empty group animals at dpi 3, while induction of IL-6 (Saline group: *p <*0.0001) and IL-10 (Saline group: *p <*0.0001; AdC68-empty group: *p <*0.001) at dpi 7 was maintained. In the vaccinated animals (low- and high-dose vaccine groups), IFN-γ, IL-6 and IL-10 levels measured at both time points were not significantly different from those of the negative control group. The levels of IL-2, IL-5, IL-12p40 and IL-21 in animals of the Saline group and AdC68-empty group showed a trend towards increased lung expression at dpi 3, but these values were not statistically significant. The levels of TNF-α, IL-4 and IL-17 did not show any significant sign of induction after SARS-CoV-2 infection.

Moreover, mean histopathology scores ≥3 in animals in the Saline group and AdC68-empty group were dramatically higher than those in the negative control group (mean histopathology score of 3 animals was below 0.1). BV-AdCoV-1 vaccination markedly reduced mean histopathology scores (1.08 and 1.38, respectively) in the lungs of the low-dose and high-dose vaccine groups at dpi 7 (*p <*0.01), respectively (**Figure 6C**). BV-AdCoV-1 vaccination reduced lung inflammation (as evidenced by reduced inflammatory leucocyte infiltrates in the lung tissue) and edema (tissue swelling) in golden Syrian hamsters challenged with SARS-CoV-2 (**Supplementary Material 2**).

Overall, vaccination with BV-AdCoV-1 could protect the lungs of immunized golden Syrian hamsters from an infectious SARS-CoV-2 challenge and induces cross-neutralizing antibody protection against WT, and the Delta, Omicron (BA.1), Omicron (BA.2.75) and Omicron (BA.4/5) variants of concern.

## Discussion

In spite of the remarkable efficacy of approved injectable COVID-19 vaccines which induce neutralizing antibodies, elicit polyfunctional T-cell responses and confer protection, new-generations of vaccines stimulating the mucosa-associated lymphoid tissue (MALT) are needed to provide not only systemic responses but also strong long-lasting mucosal immunity to limit virus infection, minimize shedding and prevent transmission (Tiboni et al., 2021). Injectable adenovirus-based SARS-CoV-2 vaccines expressing the full-length spike have efficiently conferred protection in animals and humans (Mendonça et al., 2021). Currently, several adenovirus-vectored SARS-CoV-2 vaccines are being evaluated for their ability to elicit mucosal immunity in the upper and lower respiratory tracts using intranasal administration (Dhama et al., 2022).

In this study, we report that intranasal vaccination of golden Syrian hamsters with BV-AdCoV-1, a chimpanzee adenovirus-vectored vaccine expressing a stabilized SARS-CoV-2 pre-fusion S-2P protein confers immunoprotection against live SARS-CoV-2 challenge and elicits broad cross-neutralizing antibody against prevalent epidemic strains, including the current Omicron (BA.1), Omicron (BA.2.75) and Omicron (BA.4/5) variants of concern. The Syrian hamster model has proved to be a valuable model to evaluate SARS-CoV-2 pre-S and full-length S-based vaccines (Hassan et al., 2020; Doremalen et al., 2021; Lubbe et al., 2021; Boudewijns et al., 2022). A stabilized pre-S spike was used as immunogen since it was reported that the MERS S-2P protein was better expressed and more immunogenic than the S monomer and its wild-type spike counterpart, probably through the preservation of conformational and quaternary neutralization epitopes (Pallesen et al., 2017). This finding was confirmed by Liu et al. (Liu et al., 2021) who showed that a single intranasal injection of a live-attenuated parainfluenza virus-vectored SARS-CoV-2 pre-S-2P induced significantly higher levels of neutralizing antibodies than the SARS-CoV-2 spike. A chimpanzee adenovirus vector was preferred over human adenovirus vectors because of the very low prevalence of pre-existing anti-vector antibodies in humans (Xiang et al., 2006). The results indicated that intranasal delivery of BV-AdCoV-1 elicited robust humoral and cell-mediated responses. Two doses of vaccines induced high levels of S-, S1- and RBD-specific serum antibodies capable of neutralizing SARS-CoV-2 even at the low dose of BV-AdCoV-1 (3.4×10^9^ VP). Neutralizing titers were equivalent to those obtained with the NIBSC 20/136 reference or following intranasal immunization of Syrian hamsters with the ChAdOx1 nCoV-19 vaccine which expresses a full-length spike (Doremalen et al., 2021). The results showed that BV-AdCoV-1 could significantly reduce lung viral load (*p* =0.0067) and pathology (*p <*0.0001) in immune hamster just as well as the ChAdOx1 nCoV-19 vaccine, that could significantly reduce lung viral load (*p* =0.0068) while inducing almost no lung pathology. The ChAdOx1 nCoV-19 vaccine also reduced shedding and prevented hamster-to-hamster transmission. A single intranasal dose of another chimpanzee vector expressing the pre-fusion spike induced high levels of neutralizing antibodies, systemic and mucosal IgA responses, as well as T-cell responses and almost prevented infection in mice (Hassan et al., 2020). Furthermore, a recent study comparing the hAd5-nCoV vaccine delivery either through aerosol inhalation or intramuscular administration showed that inhalation was safe and required only a 1/5 to 2/5 of the intramuscular dose to achieve equivalent immunogenicity (Wu et al., 2021).

Moreover, BV-AdCoV-1 elicited S-specific T-cell responses as judged by IFN-γ ELISpot analysis of immune splenocytes. Intranasal vaccination significantly reduced weight loss in animals following SARS-CoV-2 infection. In addition, immunization with BV-AdCoV-1 also markedly reduced relative levels of RNA in the lungs, SARS-CoV-2 infectious titers and lung pathology scored on lung inflammation, edema and hemorrhage. SARS-CoV-2-induced lung pathology in hamsters appeared to be driven by immune pathology, as lung injury at 4 dpi was markedly reduced in STAT2 knockout hamsters (Boudewijns et al., 2020). Chan et al. reported that expression of TNF-α, IFN-γ and other proinflammatory cytokines such as IL-2, IL-5, IL-12p40 and IL-21 usually peaked at dpi 3 in the lungs of infected hamsters (Chan et al., 2020). Here, we observed that IFN-γ, IL-6 and IL-10 RNA levels were significantly reduced in BV-AdCoV-1-immunized animals, going down to baseline levels observed in non-infected animals. Vaccinated golden Syrian hamsters were thus fully protected against a live SARS-CoV-2 challenge. In addition, histopathology results showed that the BV-AdCoV-1 vaccine did not induce enhanced disease nor immunopathological exacerbation.

It has been reported that a single intranasal dose of COVID-19 vaccine could induce neutralizing antibodies and protect against SARS-CoV-2 infection in pre-clinical models (Doremalen et al., 2021; Liu et al., 2021). In this study, both a low and high booster dose of vaccine significantly enhanced virus neutralizing antibody titers, indicating that a two-dose vaccination schedule will yield optimal immunogenicity results.

Compared to systemic immunization, vaccines administered mucosally not only elicit humoral immunity but also local antibodies, including sIgA, and cellular responses that are more efficient at protecting the lower and upper respiratory tracts against infection, limiting shedding and preventing virus transmission. In addition, mucosal vaccine delivery is non-invasive, convenient, easily accessible and thus, represents a promising approach to facilitate mass immunization, overcome vaccine hesitancy and achieve herd immunity. Currently, several mucosal COVID-19 vaccines administered *via* nasal spray or aerosols are being evaluated in particular by Bharat Biotech (India) and Wantai BioPharm (China). Vaccination by inhalation has been successfully used for influenza, MMR (measles, mumps, rubella) and human papillomavirus (HPV) vaccines (Bennett et al., 2002; Nardelli-Haefliger et al., 2005; Amorij et al., 2010).

In our study, no enhanced infection, immunopathology, or disease was observed in immunized Syrian hamsters challenged with live virus. Notably, a two-dose immunization regimen with BV-AdCoV-1 could protect the lungs of immunized hamsters from infectious SARS-CoV-2 challenge and induced broad cross-neutralizing antibody responses against epidemic variants of concern. Based on these preclinical data, we suggest that mucosal delivery *via* nasal spray or aerosolization of BV-AdCoV-1, is a promising platform to safely prevent SARS-CoV-2 infection, disease, and transmission, and warrants further evaluation in humans as a primary or heterologous booster immunization strategy.

## Data availability statement

The raw data supporting the conclusions of this article will be made available by the authors, without undue reservation.

## Ethics statement

The animal study was reviewed and approved by the Institutional Animal Care and Use Committee of Oncodesign (CNREEA Agreement N° 91) and the CEA (Commissariat à lEnergie Atomique; CETEA DSV n° 44) and Wuhan Myhalic Biotechnological Co., Ltd(No. HLK-20220630-001).

## Author contributions

SW, LXu, MQ, and LD designed the experiment protocol. TM and LXi designed and produced BV-AdCoV-1. PZ and YW coordinated the projects. NL, KM, and GF performed the challenge experiments and data analysis. YL and WY identified and analyzed the purified pre-S protein, while JZ, MJ, and BG performed the immunogenicity study in China. SW and LXu wrote the manuscript. MK and KW supervised the research and finalized the manuscript. All authors contributed to the article and approved the submitted version.

## Funding

This work was funded by Wuhan BravoVax Co., Ltd. and Shanghai BravoVax CO., Ltd.

## Acknowledgments

The authors wish to thank the innovative discovery department from Wuhan BravoVax Co., Ltd. for designing, engineering and producing BV-AdCoV-1.

## Conflict of interest

Author SW, LXu, TM, MQ, PZ, LXi, YW, YL, WY, JZ, MJ, BG, MK, and KW are employed by Wuhan BravoVax Co., Ltd. MK and KW are also employed by Shanghai BravoVax Co., Ltd. Meanwhile, YL is a teacher from Hubei University. LD is employed by Voisin Consulting Life Sciences. NL, KM, and GF are employed by Oncodesign.

The remaining authors declare that the research was conducted in the absence of any commercial or financial relationships that could be construed as a potential conflict of interest.

## Publisher’s note

All claims expressed in this article are solely those of the authors and do not necessarily represent those of their affiliated organizations, or those of the publisher, the editors and the reviewers. Any product that may be evaluated in this article, or claim that may be made by its manufacturer, is not guaranteed or endorsed by the publisher.

## Supplementary material

Supplementary material 1 Molecular weight of spike. **(A)** Molecular weight of spike secreted in infected HEK293A culture supernatants calculated using the Quantity One software. **(B)** Molecular weight of spike in infected HEK293A cell lysates calculated using the Quantity One software.

Supplementary material 2 Histopathological Hematoxylin-Phloxine evaluation of SARS-CoV-2 infection in golden Syrian hamsters (Hematoxylin-Phloxine 10x, scale bar = 250 µm). Pictures are shown for one animal of each group **(A)** Negative control; **(B, C)** Saline; **(D, E)** AdC68-empty; **(F, G)** Low-dose vaccine; and **(H, I)** High-dose vaccine. Arrows show signs of inflammatory leucocyte infiltrates (vertical arrows), pulmonary edema (horizontal arrows) and alveolar hemorrhage (angled arrows). Left lung slides were stained with Hematoxylin-Phloxine to visualize histomorphometric changes. Slides were scanned using the NanoZoomer Digital Pathology System C9600-02.
