# Supplementary figures and images for: Intranasal delivery of a chimpanzee adenovirus vector expressing a pre-fusion spike (BV-AdCoV-1) protects golden Syrian hamsters against SARS-CoV-2 infection
Source: Front Cell Infect Microbiol. 2022 Nov 3;12:979641. doi: 10.3389/fcimb.2022.979641 (PMC9671113; doi:10.3389/fcimb.2022.979641)

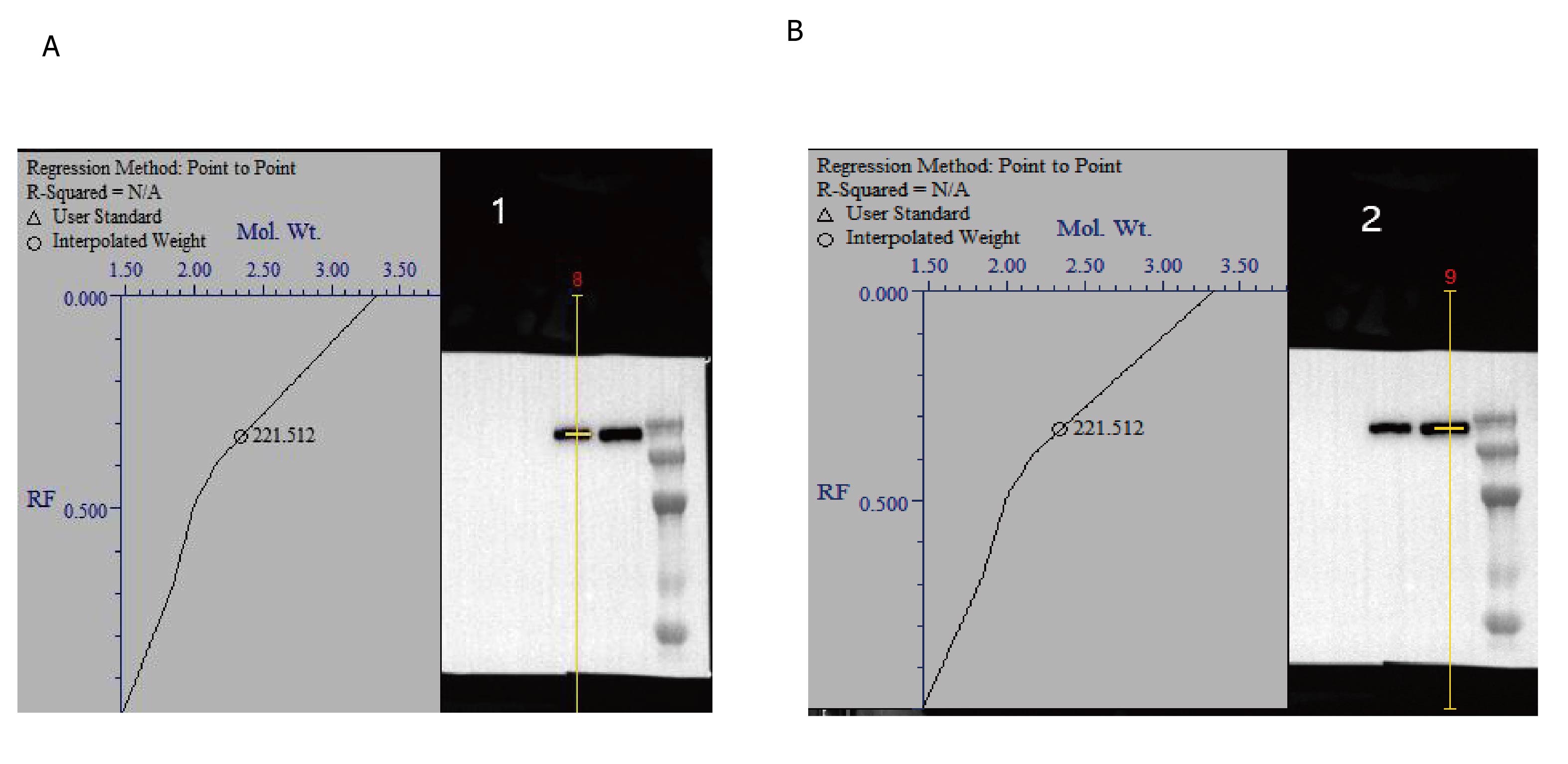

Supplement: Supplementary material 1 — Molecular weight of spike. (A) Molecular weight of spike secreted in infected HEK293A culture supernatants calculated using the Quantity One software. (B) Molecular weight of spike in infected HEK293A cell lysates calculated using the Quantity One software. [file Image_1.jpeg]

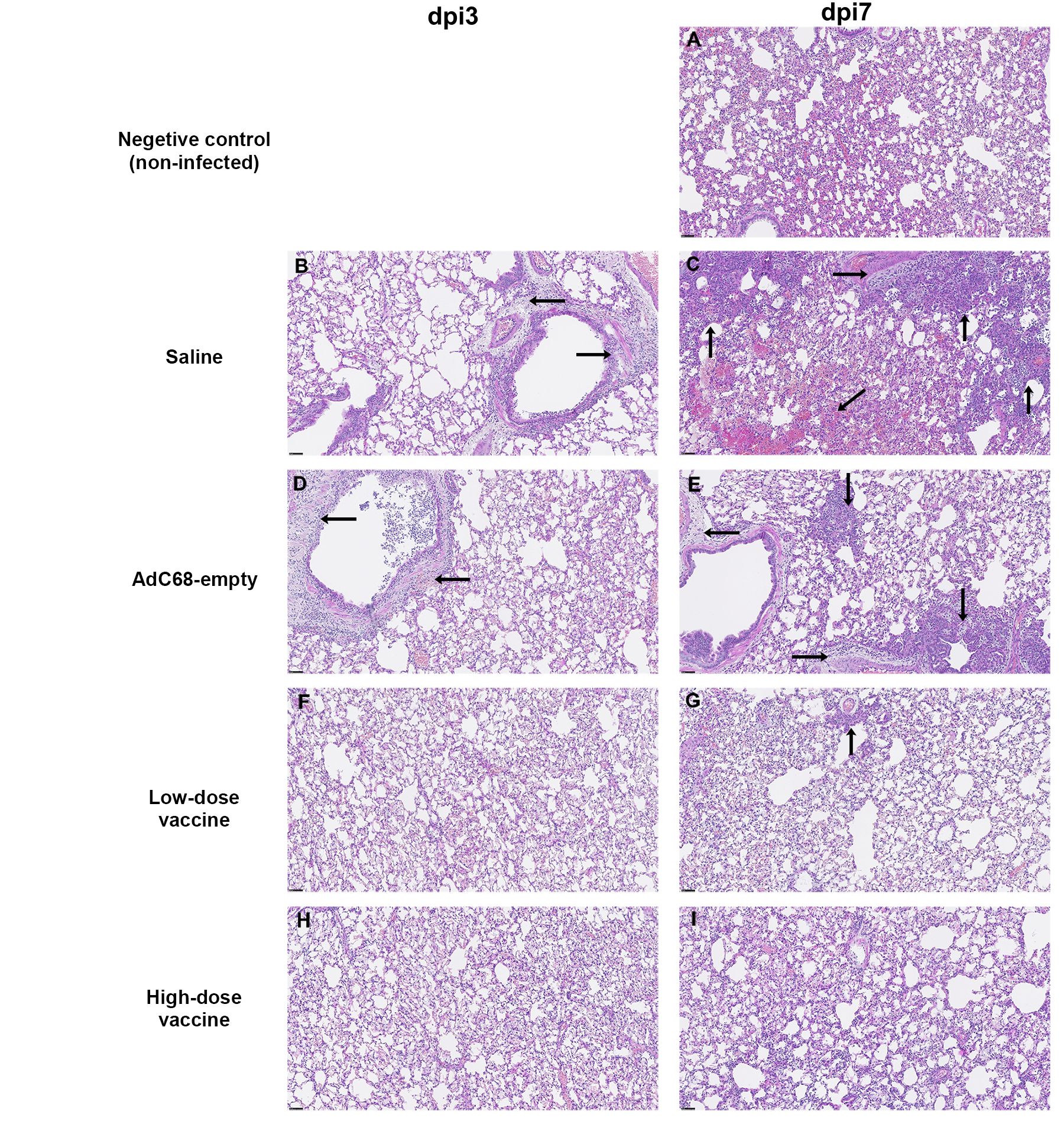

Supplement: Supplementary material 2 — Histopathological Hematoxylin-Phloxine evaluation of SARS-CoV-2 infection in golden Syrian hamsters (Hematoxylin-Phloxine 10x, scale bar = 250 µm). Pictures are shown for one animal of each group (A) Negative control; (B, C) Saline; (D, E) AdC68-empty; (F, G) Low-dose vaccine; and (H, I) High-dose vaccine. Arrows show signs of inflammatory leucocyte infiltrates (vertical arrows), pulmonary edema (horizontal arrows) and alveolar hemorrhage (angled arrows). Left lung slides were stained with Hematoxylin-Phloxine to visualize histomorphometric changes. Slides were scanned using the NanoZoomer Digital Pathology System C9600-02. [file Image_2.jpeg]
